# What evidence exists on salmon farming? An overview of reviews

**DOI:** 10.1186/s13750-026-00391-y

**Published:** 2026-07-15

**Authors:** Neal Haddaway, María D. López Rodríguez, Hazel Cooley, Matthew Grainger

**Affiliations:** 1 University of Johannesburg, Johannesburg, South Africa; 2 University of Almería, Carr. Sacramento, S/N, 04120 La Cañada, Almería, Spain; 3 Freelance researcher, Newcastle, UK; 4 Norwegian Institute for Nature Research (NINA), Trondheim, Norway

**Keywords:** Mariculture, Aquaculture, Systematic reviews, Salmo, Salmonid, Oncorhynchus

## Abstract

**Background:**

The global expansion of salmon aquaculture has transformed marine food systems, offering economic benefits while amplifying environmental and socio-economic challenges. The shift from wild-caught to farmed salmon has led to widespread concerns, including disease transmission, genetic introgression, pollution, and conflicts with Indigenous communities. Additionally, salmon feed supply chains have global implications, such as overfishing in West Africa. Although many studies exist, research is fragmented and dispersed across disciplines, and the influence of industry funding raises questions about bias. This overview of reviews synthesises existing systematic reviews on salmon farming to evaluate the scope, quality, and findings of the secondary literature on its environmental, economic, and social impacts. Specifically, we ask the question: “What systematic review evidence exists on Atlantic and Pacific salmon aquaculture, and what are the characteristics, thematic focus, and methodological quality of this evidence?”.

**Methods:**

This overview of reviews follows guidelines from the Collaboration for Environmental Evidence (CEE) and the ROSES reporting standards. Systematic reviews (broadly defined as reviews either claiming to or actually using some form of systematic methodology) were identified via searches in Scopus, Web of Science, and Lens.org using a structured, validated search strategy. Inclusion criteria focused on reviews explicitly targeting Atlantic or Pacific salmon aquaculture (including rainbow trout). Metadata were extracted on study methods, topical focus, production stages, and funding sources. Review quality was assessed using the CEESAT critical appraisal tool, and visualisations were produced to identify gaps in coverage and assess methodological rigour.

**Review findings:**

Out of 327 unique records, 37 reviews were included. Most reviews focused on production-related topics (e.g. fish health, sea lice, feed supply), with very few addressing environmental or social impacts. No reviews met the threshold for high methodological rigour, and only four were rated as moderate quality; the remainder were judged as low or exceptionally low quality. Common weaknesses included absence of a priori protocols, lack of critical appraisal, poor reporting of screening and extraction methods, and inappropriate synthesis techniques such as vote counting. Industry co-authorship was present in 7 reviews, and 8 were industry-funded. Research on sustainability was hindered by methodological inconsistency and limited transparency.

**Conclusions:**

Despite a growing body of literature on salmon aquaculture, high-quality evidence syntheses remain rare. Most systematic reviews are methodologically flawed and overly focused on productivity, with limited assessment of environmental or socio-economic externalities. Greater attention is urgently needed to unbiased, interdisciplinary, and high-quality evidence synthesis—especially on the broader impacts of salmon farming. Decision-makers should interpret existing evidence syntheses on salmon aquaculture with caution, given the generally low methodological rigour and high risk of bias identified across reviews. Apparent consensus or patterns in the literature may reflect methodological artefacts rather than robust underlying evidence. We therefore call for enhanced rigour in review methods, transparent reporting, and increased scrutiny of industry influence on research agendas.

**Supplementary Information:**

The online version contains supplementary material available at https://doi.org/10.1186/s13750-026-00391-y.

## Background

The global expansion of salmon aquaculture has reshaped marine food systems, creating economic opportunities while also raising significant environmental and socio-economic concerns. Historically, wild salmon fisheries sustained Indigenous communities, supported commercial and recreational fishing, and played a vital role in regional food security [31, 36]. However, due to declining wild populations—both Atlantic salmon [17] and Pacific salmon [30, 39]—and the increasing commercial viability of aquaculture, salmon farming has emerged as a dominant force within the seafood sector. Today, Atlantic salmon dominate global aquaculture production, with Norway, Chile, Canada, and Scotland leading production efforts [33, 38, 42]. Notably, salmon farming now occurs on every continent except Africa [3].

### Industry growth and economic contributions

Salmon aquaculture has expanded rapidly since the 1980s, driven by technological advancements, shifting market dynamics, and the increasing global demand for fish protein [32]. Norway's salmon exports, valued at 17.7 billion USD in 2022, illustrate the industry’s significance [48]. Chile, despite lacking native salmon populations, has become a key global producer, exceeding one million tonnes of production in 2020 [9]. While proponents argue that farmed salmon contributes to food security [3], critics highlight its primary consumption as a luxury product in wealthier nations, questioning its role in addressing global protein needs [7].

### Environmental and ecological concerns

The intensification of salmon farming has spurred widespread environmental concerns that vary across the key producing countries. Key issues include disease transmission to wild populations [28, 45], high mortality rates linked to parasites and environmental stressors [4, 40, 41], and habitat degradation caused by nutrient pollution [8]. Additionally, escapes of farmed salmon threaten the genetic integrity of wild stocks [10], while antibiotic overuse raises concerns about antimicrobial resistance [34]. Further downstream impacts stem from feed supply chains, including the depletion of West African fisheries for fishmeal and fish oil [21] and the deforestation associated with soy production for feed [1].

### Climate change and sustainability challenges

Climate change presents a growing challenge to salmon aquaculture. Rising ocean temperatures exacerbate disease susceptibility [12] and disrupt production through extreme weather events, increasing infrastructure vulnerability and plastic pollution [20, 23]. Ocean acidification further threatens the industry by altering salmon physiology and food web dynamics [43]. While industry stakeholders seek adaptive strategies, the long-term sustainability of salmon farming remains a contentious issue, with evidence suggesting failures in environmental and welfare certification schemes [19].

### Social and regulatory dimensions

Beyond environmental concerns, salmon farming has profound socio-economic implications. Conflicts with Indigenous and artisanal fishing communities continue to escalate [27, 46], while opposition to farm expansion grows due to concerns over noise, odour, and ecosystem impacts [29, 51]. Occupational hazards within the industry have also drawn scrutiny, particularly following recent worker fatalities [5]. Regulatory frameworks vary by region, with certification schemes such as the Aquaculture Stewardship Council (ASC) and the Marine Stewardship Council (MSC) aiming to ensure sustainability [47]. However, concerns persist regarding industry influence over regulatory bodies and certification processes [18].

### Industry transparency and public accountability

The salmon industry’s approach to scrutiny has raised critical questions about transparency and accountability. Journalists and activists investigating environmental and welfare issues frequently face legal challenges and restricted access, as exemplified by cases involving Don Staniford [49]. Investigations have uncovered falsified records relating to animal welfare and environmental standards [22, 50], underscoring the need for independent oversight.

### The role of research: identifying biases and knowledge gaps

Academic research on salmon farming is extensive yet often fragmented and dispersed across disciplines, limiting holistic understanding. Funding from industry stakeholders poses a risk of bias, shaping research agendas and outcomes in ways that may favour commercial interests. Preliminary analysis indicates that 7.4% of funding statements in a Lens.org search explicitly reference industry funding, though 90% lack indexed funding disclosures. This highlights the necessity of synthesising independent research to provide a balanced assessment of the environmental, economic, and social ramifications of salmon farming.

### Rationale for an overview of reviews and stakeholder engagement

Despite the breadth of literature on salmon farming, there remains a lack of comprehensive synthesis. Existing reviews often focus on narrow geographical regions e.g., [45] or adopt narrative rather than systematic approaches e.g., [16]. An overview of reviews consolidating systematic reviews across multiple domains is needed to bridge these gaps, map research effort across economic, environmental, and social dimensions, and inform evidence-based policy and practice. This review aims to provide an integrated perspective on salmon farming’s sustainability, highlighting areas requiring further investigation and independent scrutiny.

Key informant interviews were conducted during question formulation with individuals and groups involved in salmon farming research and activism, including representatives of NGOs, members of activist organisations, journalists, independent researchers, and government-funded research institutes, aiming to ensure shared definitions of the species included.

## Objective of the review

The aim of this review was to assess the body of secondary research focusing on salmon (Atlantic and Pacific) farming, understanding the topics covered and quality of the syntheses.

**Primary question: **What systematic review evidence exists on Atlantic and Pacific salmon aquaculture, and what are the characteristics, thematic focus, and methodological quality of this evidence?

The review has the following key components:

**Population: **Aquaculture worldwide.

**Phenomenon:** Atlantic salmon (*Salmo salar*), as well as Pacific salmon that includes several species commonly referred to as salmon—i.e. steelhead salmon (*Oncorhynchus mykiss*, including the rainbow trout which is a form that solely resides in freshwater), coho salmon (*Oncorhynchus kisutch*), chinook salmon (*Oncorhynchus tshawytscha*), pink salmon (*Oncorhynchus gorbuscha*), sockeye salmon (*Oncorhynchus nerka*), masu salmon (*Oncorhynchus masou*) and chum salmon (*Oncorhynchus keta*).

**Study design:** Literature reviews claiming to be a “systematic review” or using one or more systematised aspects of methodology, from searching through to synthesis.

For the scope of this review, we wanted to ensure we had a consistent system boundary with regards to systematic reviews. Despite many robust and rigorous definitions existing for systematic reviews published by internationally accepted organisations e.g. [14], substandard reviews that do not meet the requirements of these definitions continue to be published, and many unreliable reviews refer to themselves explicitly as being ‘systematic’. As such, we wanted to ensure a broader definition of the term ‘systematic review’ to encompass *both* reviews that organisations such as CEE would recognise *and* reviews that make claims to be but are far from best practice. Martinic et al. [35] define a systematic review as a literature review that includes: (1) a research question, (ii) sources that were searched, with a reproducible search strategy (naming of databases, naming of search platforms/engines, search date and complete search strategy); (iii) inclusion and exclusion criteria; (iv) selection (screening) methods; (v) critically appraises and reports the quality/risk of bias of the included studies; and, (vi) information about data analysis and synthesis that allows the reproducibility of the results. Although we agree with this definition for strict systematic reviews, its application in overview of reviews outside the field of healthcare would yield very little admissible evidence, for example also excluding systematic maps.

In order to reflect on the broader quality and usability of evidence synthesis in the field, our definition of a ‘systematic review’ broadens this strict definition to include any review that claims to be a systematic review either by name (i.e. ‘systematic review’ or ‘systematic literature review’) or that reports the use of some aspect of formal methodology in conducting searching, screening, data extraction, appraisal or synthesis. As such, we also include any review that conducts quantitative synthesis on the understanding that comprehensiveness is vital to minimising the risk of bias in the findings. We also note that evidence synthesis in the field of environment often uses diverse methods that do not conform to strict definitions taken from health, including systematic maps, for example. Our definition is inclusive to these review types. By adopting an inclusive definition of “systematic review”, we necessarily include studies that may not fully adhere to established methodological expectations for systematic evidence synthesis. Evaluating these studies against such standards may therefore be seen as methodologically inconsistent. However, we consider this an inherent feature rather than a flaw of our approach: our aim is not only to assess those reviews that meet idealised definitions, but to evaluate how the term “systematic review” is currently used in the included literature, and the extent to which this usage corresponds to recognised principles of rigorous evidence synthesis. In this sense, any mismatch between claims of systematicity and observed methodological practice is itself a key finding of the review.

## Methods

This overview was conducted according to an a priori published protocol [25] and follows the general guidance for evidence reviews outlined by the Collaboration for Environmental Evidence [14]. The review is reported according to the ROSES reporting standards for evidence syntheses [24]. There are no deviations from the protocol.

We conducted an overview of reviews (also referred to as an “umbrella review”, “review of reviews”, “synthesis of systematic reviews”, “summary of systematic reviews”, “evidence review mapping” or “systematic map of reviews”), defined as a systematic synthesis of evidence from multiple systematic reviews on a shared topic [2]. Unlike a conventional systematic review, the unit of inclusion and analysis is the review rather than the primary studies within those reviews [6]. We refer to literature reviews as “reviews”, while the primary research contained within these reviews is referred to as “studies”. Where multiple reviews are reported within a single document, these are treated as distinct reviews, and the document is referred to as an “article”. During screening, we refer to ‘records’ being the bibliographic data points that are assessed for eligibility.

### Search for articles

#### Bibliographic database searches

We searched for systematic reviews across ten databases via three bibliographic resources, Web of Science (including Core Collections (1900–2025), Current Contents Connect (1998–2025), Derwent Innovations Index (1980–2009), Grants Index (1953–2025), KCI-Korean Journals Database (1980–2025), MEDLINE (1950–2025), ProQuest Dissertations & Theses (1637–2025), and SciELO (2002–2025), searched on 16th February 2025)), Lens.org and Scopus (both searched on 18th February 2025). We used the search string outlined in Table 1 and Additional file 1. Since we focus on academic research literature, we searched only in English.

**Table 1:** Bibliographic database search strings

Substring	String terms
#1	title/abstract/keywords: (salmon OR salmo OR oncorhynchus OR salmonid OR trout OR salmonid OR salmoniformes)
#2	title/abstract/keywords: (aquaculture OR mariculture OR *cultur* OR farm* OR rearing)
#3	title/abstract/keywords: ("scoping review" OR "literature review" OR "systematic review" OR "systematic map" OR "systematic mapping" OR "systematically review" OR "systematically reviewed" OR "review of the literature" OR "evidence review" OR "review of the evidence" OR "rapid evidence assessment" OR "rapid review" OR "evidence synthesis" OR "systematic literature" OR "review of the literature")
#4	title: (salmon OR salmo OR oncorhynchus OR salmonid OR trout OR salmonid OR salmoniformes) AND (aquaculture OR mariculture OR *cultur* OR farm* OR rearing) AND (“a review”)
#5	(#1 AND #2 AND #3) OR #4

Search results were exported and combined before being deduplicated first in EndNote using the method outlined by Bramer et al. [11] and then in the web application Rayyan.ai. During deduplication in EndNote, clearly irrelevant titles that were not research articles (e.g. reference lists or book reviews) were removed prior to exporting the results to Rayyan. The subsequent unique results were then screened as outlined below.

##### Testing comprehensiveness of the search

In order to assess the comprehensiveness of our search string we assembled a list of 6 reviews of known relevance through consultations with stakeholders and analysis of the bibliographies of relevant reviews encountered during background research. We then identified whether our string returned these records, which it did, indicating sufficiency. This benchmark list of records is provided in the Additional file 5.

##### Citation chasing

We performed forward (citing records) and backward (cited records) citation chasing using citationchaser [26], based on the final set of included reviews possessing DOIs (n = 35), yielding 3,439 records from backwards citation chasing and 864 records from forward citation chasing. These results were then filtered by the review terms in line 3 of the search string above to retain only those records that were reviews. The resulting records were deduplicated against the original search results, and the remaining unique records were screened as outlined below.

##### Search update

Since this overview of reviews was completed within a short period (searches conducted in February 2025) and the publication rate for the field is very low, we did not plan to conduct a search update.

### Article screening and study eligibility criteria

#### Screening process

We screened records at two levels: title and abstract, and full text. We performed an initial consistency check with 50 titles and abstracts, and 5 full texts to ascertain reviewer agreement between two reviewers (NRH and MDLR) and ability to use the inclusion criteria. At title and abstract level, an initial screening of 50 records revealed 7 conflicts, which were resolved immediately on discussion. All excludes from the initial screening (by NRH) were then double checked (by MDLR). This resulted in 11 conflicts, which were then appraised (by NRH) at full text, with no conflicts requiring inclusion. At full text screening, a random subset of 18 records (10%) was screened blind by both reviewers. A single conflict occurred but was immediately resolved on clarification of the type of review involved (finally excluded). Following consistency checking, screening was then performed by one review (NRH), with discussions across the review team where cases appeared uncertain. The fates of all screened records at full text assessment are provided in Additional file 2.

##### Eligibility criteria

We used the following inclusion criteria:

**Population: ** Aquaculture worldwide.

**Phenomenon:** Atlantic salmon (*Salmo salar*), which forms 90% of farmed salmon across the world, as well as Pacific salmon that includes several species commonly referred to as salmon—i.e. steelhead salmon (*Oncorhynchus mykiss,* including the rainbow trout which is a form that solely resides in freshwater), coho salmon (*Oncorhynchus kisutch*), chinook salmon (*Oncorhynchus tshawytscha*), pink salmon (*Oncorhynchus gorbuscha*), sockeye salmon (*Oncorhynchus nerka*), masu salmon (*Oncorhynchus masou*) and chum salmon (*Oncorhynchus keta*). Salmon had to be a core focus of the review, either within the search string or an explicit a priori focus. We thus excluded studies where relevant salmon species were just one of a number of other species incidentally reported within the abstract (and results).

**Study design:** Reviews were eligible if they (i) self-identified using terms such as “systematic review”, “scoping review”, “evidence synthesis” (see substring #3 in our search above for a comprehensive list), (ii) reported the methods for at least one explicit methodological component of systematic evidence synthesis (e.g. structured searching, screening, data extraction, critical appraisal, or quantitative synthesis), or (iii) presented a quantitative synthesis across studies (i.e. meta-analysis or allegorical method) that warrants a systematic methodological approach. Reviews that did not self-identify as systematic, did not report any explicit methodological components, or did not perform quantitative synthesis without any methodology were excluded.

None of the authors of this review were involved with the conduct of any included literature reviews, meaning that preventing decisions on our own work was not a relevant concern.

### Assessing study reliability

We used the CEESAT critical appraisal tool [14] for assessing evidence review methodological quality, which involves 16 questions about the methods used across planning, searching, screening, data extraction, critical appraisal and synthesis of systematic reviews. The CEESAT tool assigns a grade based on explicit criteria for each question, resulting in judgements of red, amber, green or gold. All reviews were assessed by two reviewers (NRH and HC) independently, before all discrepancies were discussed in detail and an agreement made on the final score for each question.

We made the following minor modifications to CEESAT to reflect a more pragmatic approach to judging reliability and addressing inconsistencies in the current wording of CEESAT:

- Question 4.2 (“Are eligibility criteria consistently applied to all potentially relevant articles and studies found during the search?”)—Where 2 or more reviewers independently screened all records BUT failed to report the results of consistency checking, a judgement of “Green” was awarded (i.e. they were not penalised to “Red” for failing to report consistency if each record was screened by 2 or more reviewers). We felt this is unnecessarily penalising a method that is still more robust than single screening with no consistency checking.
- Question 7.2 (“Is a statistical estimate of pooled effect (or similar) provided together with measure of variance and heterogeneity among studies?”)—Where reviews extracted study findings and presented the direction and significance of effect but did NOT perform critical appraisal AND (even if they present magnitude of effect) they were given a “Red” judgement to account for studies that both perform and facilitate vote-counting. We felt that vote-counting was not adequately captured in CEESAT.
- Question 8.1 (“Have the authors considered limitations of the synthesis?”)—Reviews were judged as “Green” even if the limitations text focuses purely on the limitations of the review methods and there is no discussion of the limitations of the evidence base. We felt that this otherwise penalises reviews that discuss the limitations of the evidence base extensively in their results without further repetition in a dedicated section.

We then used the following criteria in Table 2 to assign a reliability judgement across all CEESAT questions for each review.

**Table 2:** Review-level reliability judgements given according to the 16 CEESAT questions for each included review

Reliability	Description
Exceptionally low	Where 10 or more questions are judged to be "Red"
Low	Where a review scores “Red” on one or more of the following critical criteria: 3.1 (Search repeatability); 3.2 (Comprehensiveness of resources); 4.1 (Clarity of eligibility criteria); 5.1 (Critical appraisal of study validity); 6.2 (Reporting extracted data)
Moderate	When a review has no “Red” judgements for critical questions (3.1, 3.2, 4.1, 5.1, 6.2) and between 1 and 4 “Red” scores for non-critical questions (50% of non-optional responses)
High	Everything else (no “Red” scores)

### Data coding strategy

We coded and extracted meta-data for all included full texts using the schema outlined in Table 3. Before coding and meta-data extraction, a subset of 3 studies was used for trial data extraction by two reviewers. Conflicts were discussed in detail but no further modifications to the schema or definitions were necessary. All relevant information was obtainable without the need to obtain data from authors.

**Table 3:** Coding and meta-data schema used on all included full texts

Field	Description
Author-year	First author surname—publication year
Title	Article title
Source-volume-pages	Journal—volume—start and end pages
Geographical focus	Countries or regions covered by the review
Authors’ method name	The type of review as described by the authors (e.g. “systematic review”)
Review type	Evidence map / Narrative synthesis / Quantitative synthesis / Qualitative synthesis (definitions following the CEE Guidelines, CEE 2022, and expanded in meta-data in Additional file 3)
Cited methodology	Authors’ reference to methodology used in the review
Species	Atlantic salmon, Rainbow trout, Coho salmon, Chinook salmon, Chum salmon, Sockeye salmon, Pink salmon, Not stated—salmon, Not stated—trout, Not stated—salmon and/or trout
Study objectives	Objectives as described by the authors
Production stage	Feed supply chain, Hatchery, Land-based grow out, Sea-transfer, Sea-cage, Harvest, In-vitro, Salmon supply chain, Consumption, Legislation and policy, Not stated
Topic (hierarchical and clustered, see Additional file 3 for clustering)	Broad topic: Business and economy, Consumption, Impacts, Production, Research methods Subtopic: Companies, Customer preference, Economy, Legislation, Public image, Supply chains, Contaminant, General, Mislabelling, Palatability, Quality, Safety, Environmental impacts, Mitigation, Social impacts, Aquaculture facility, Feed, Fish biology, Fish health, Fish welfare, Management, Pathology, Methodology, Research stage Feature: Companies, Customer preference, Economy, Legislation, Public image, Supply chains, Contaminant, General, Mislabelling, Palatability, Quality, Safety, Biodiversity impacts, Escapes, Impacts on native fish, Nutrient load, Chemicals, Wild versus farmed, Management, Output processing, Social impacts, Equipment, Hydrodynamics, Type, Additives, Alternatives, Contaminants, Conventional, Anatomy, Development, Fish behaviour, Histology, Life stage, Outcome, Reproduction, Species, Gut health, Immunity, Injury, Toxicity, Pain, Stress, Treatment, Externalities, Fish by-products, Fish identification, Practices, Production stage, Productivity, Sustainability, Illness, Impacts, Organism, Prevention, Sea lice, Literature review, Modelling, Monitoring, Research stage Component: Companies, Customer preference, Economy, Legislation, Public image, Supply chains, Contaminant, General, Mislabelling, Palatability, Quality, Safety, Biodiversity impacts, Escapes, General, Impacts on native fish, Nutrient load, Chemicals, Wild versus farmed, Escape mitigation, Waste treatment, Water treatment, Social impacts, Equipment, General, Hydrodynamics, Closed, Land-based, Location, Polyculture, Pond-based, Sea-based, Additives, Alternatives, Contaminants, Conventional, General, Management, Supply chains, Anatomy, Developmental problems, Fish behaviour, General, Histology, Life stage, Genetic modification, Genetics, Sex control, Outcome, Reproduction, Atlantic salmon, Chinook salmon, Chum salmon, Coho salmon, Pink salmon, Rainbow trout, Sockeye salmon, General, Gut health, Immune response, Injury, Toxicity, General, Pain, Stress, Anaesthetic, Stress treatment, Externalities, Fish by-products, Fish identification:, General, Farm management, Maximising productivity, Predator deterrent, Production stage, Productivity, Sustainability, General, Illness, Impacts, Organism, Antiseptic, Disinfectant, Vaccination, General, Impacts, Species, Treatment, Anti-parasitic, Antibiotic, Antibiotic resistance, Antimicrobial, General, Insecticide, Nematicide, General, Literature review, Modelling, Monitoring, Research stage
Included studies	Number of primary studies included in the review
Institute(s)	Affiliation institutions of all authors
Funder	Reported funding body/bodies

During data extraction, full texts that contained more than one review (e.g. two chapters of a thesis) were separated and are now described as ‘reviews’, whilst the document from which they came are referred to as ‘articles’.

### Data synthesis and visualisation

We synthesised and visualised the included reviews based on key variables extracted during data coding (Table 3). These included bibliographic characteristics (e.g. publication year, journal, author affiliation), review characteristics (e.g. review type, number of primary studies included), and thematic variables (e.g. species, production stage, and topic classifications). Topic classifications were structured hierarchically into broad topics (e.g. production, impacts, consumption, business and economy, research methods), subtopics, and more detailed feature and component levels (see Additional file 3).

We also visualised the methodological quality of included reviews based on CEESAT appraisal results, including both individual question scores and overall reliability categories (exceptionally low, low, moderate, high). Visualisations display the distribution of reviews across these variables and categories to identify patterns in research focus and methodological rigour.

### Deviations from the protocol

There were no deviations from the protocol.

## Review findings

### The review process

**Fig. 1:**
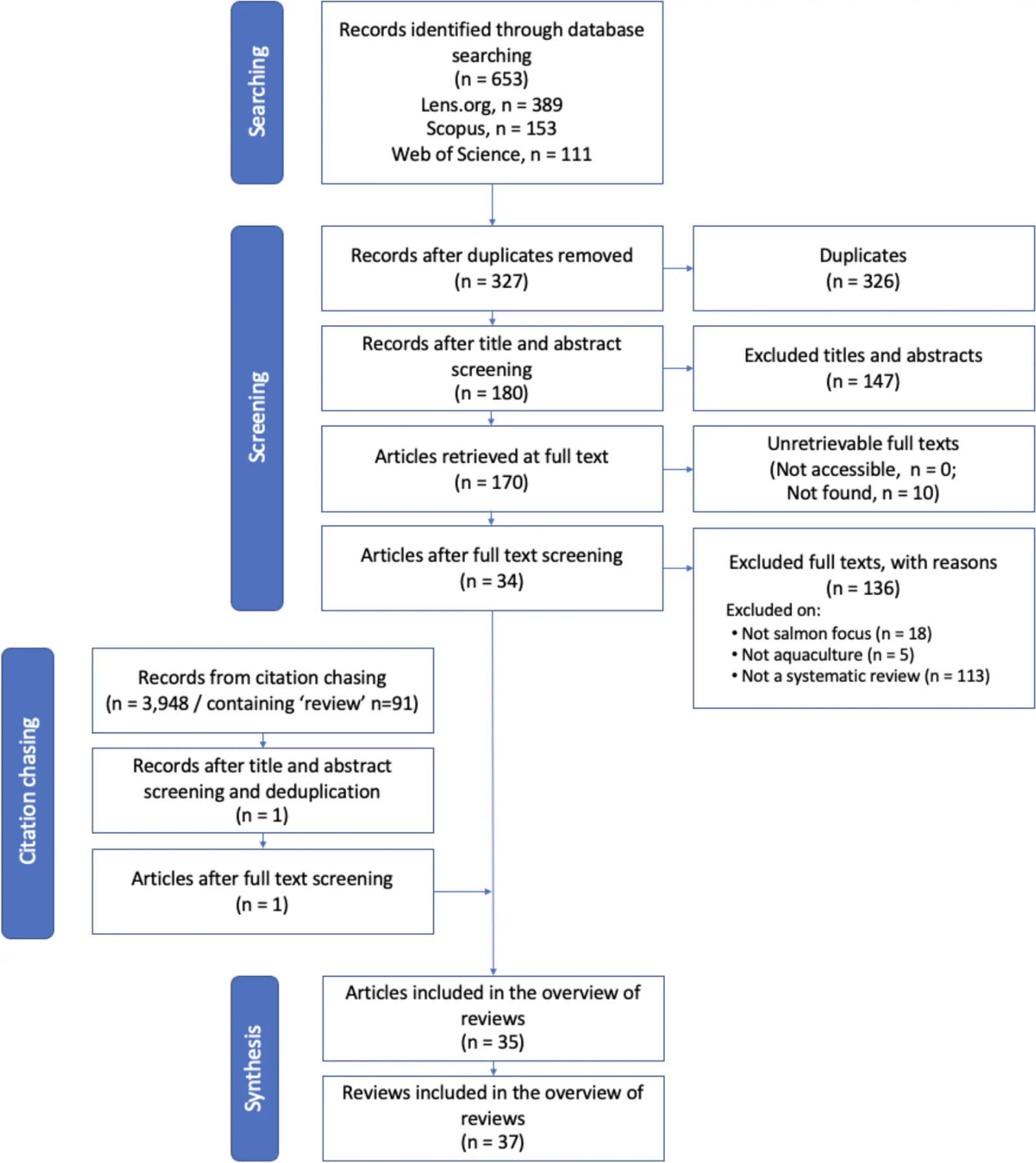
Adapted ROSES flow diagram showing fate of records through the overview of reviews process

The review process and the fate of all considered records is outlined in Fig. 1. Our database searches yielded a total of 653 results and 327 unique records. A total of 180 records were retained after title and abstract screening, with 10 unobtainable at full text and 34 records remaining after full text screening. A list of all records excluded at full text screening, with exclusion reasons, is provided in Additional file 2.

A set of 39 full texts deemed to initially be relevant were subjected to citation chasing. In all, 37 out of these 39 reviews had DOIs and were entered into citationchaser [26], yielding 3,439 records from backwards citation chasing and 864 records from forward citation chasing (3,948 unique citation chasing records). We searched within these records for any record including the word ‘review’ in its title, abstract or keywords, yielding 91 records. Finally, only one record was relevant at title and abstract and had not already been screened. This record was relevant at full text and retained, making a total of 35 records and 37 reviews included in the final database.

### The evidence base

**Fig. 2:**
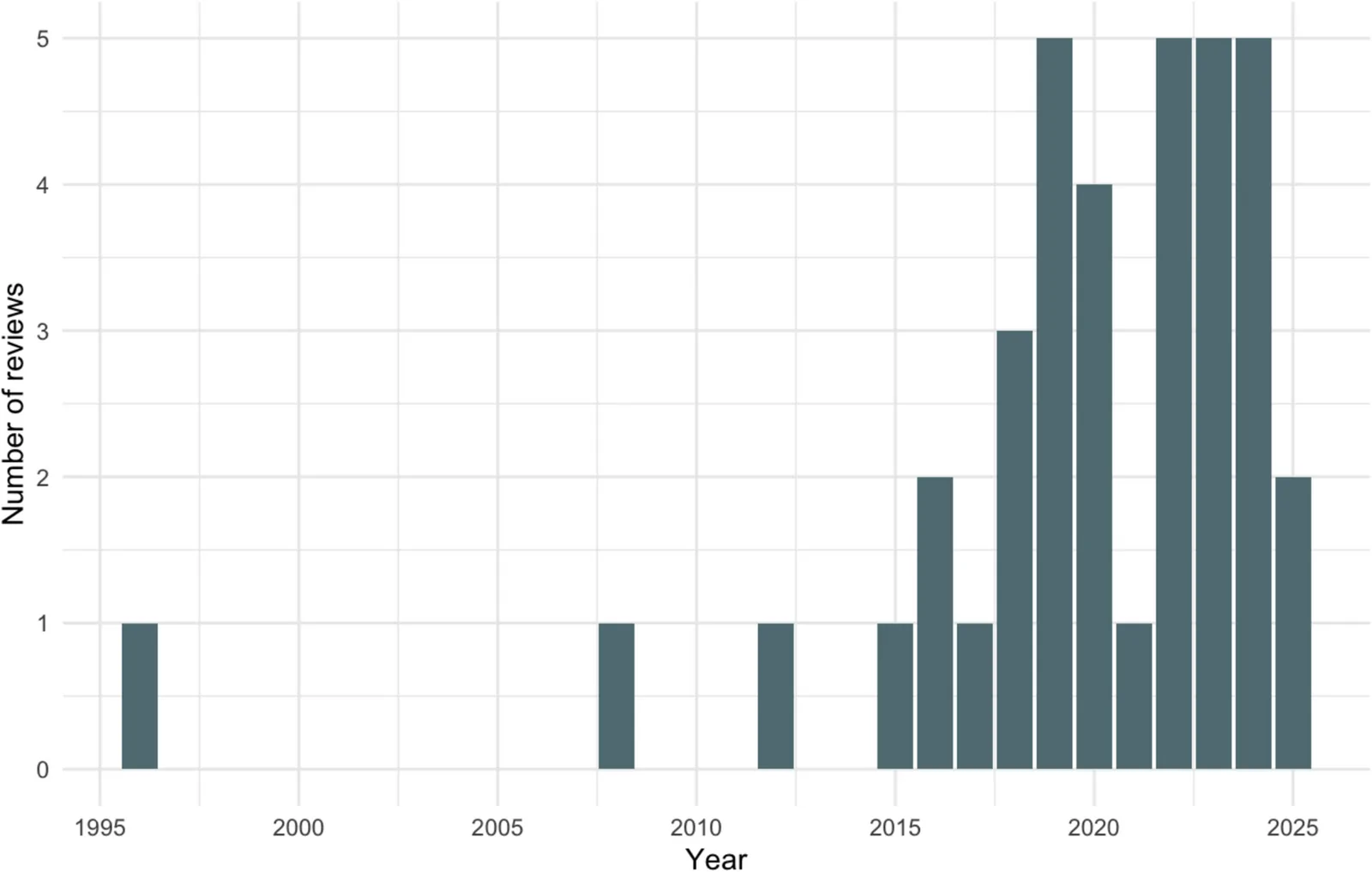
Number of included reviews by publication year

The full datasheet for all included reviews is provided in Additional file 3. Research interest via evidence synthesis has increased roughly in line with other fields and primary research in recent years (Fig. 2). The vast majority of reviews were published after 2015, with 5 reviews (14%) per year published between 2022 and 2024. One review was published in 1996, well before systematic review methods were established in the field but was included because it uses quantitative synthesis to summarise the primary studies it contained.

**Fig. 3:**
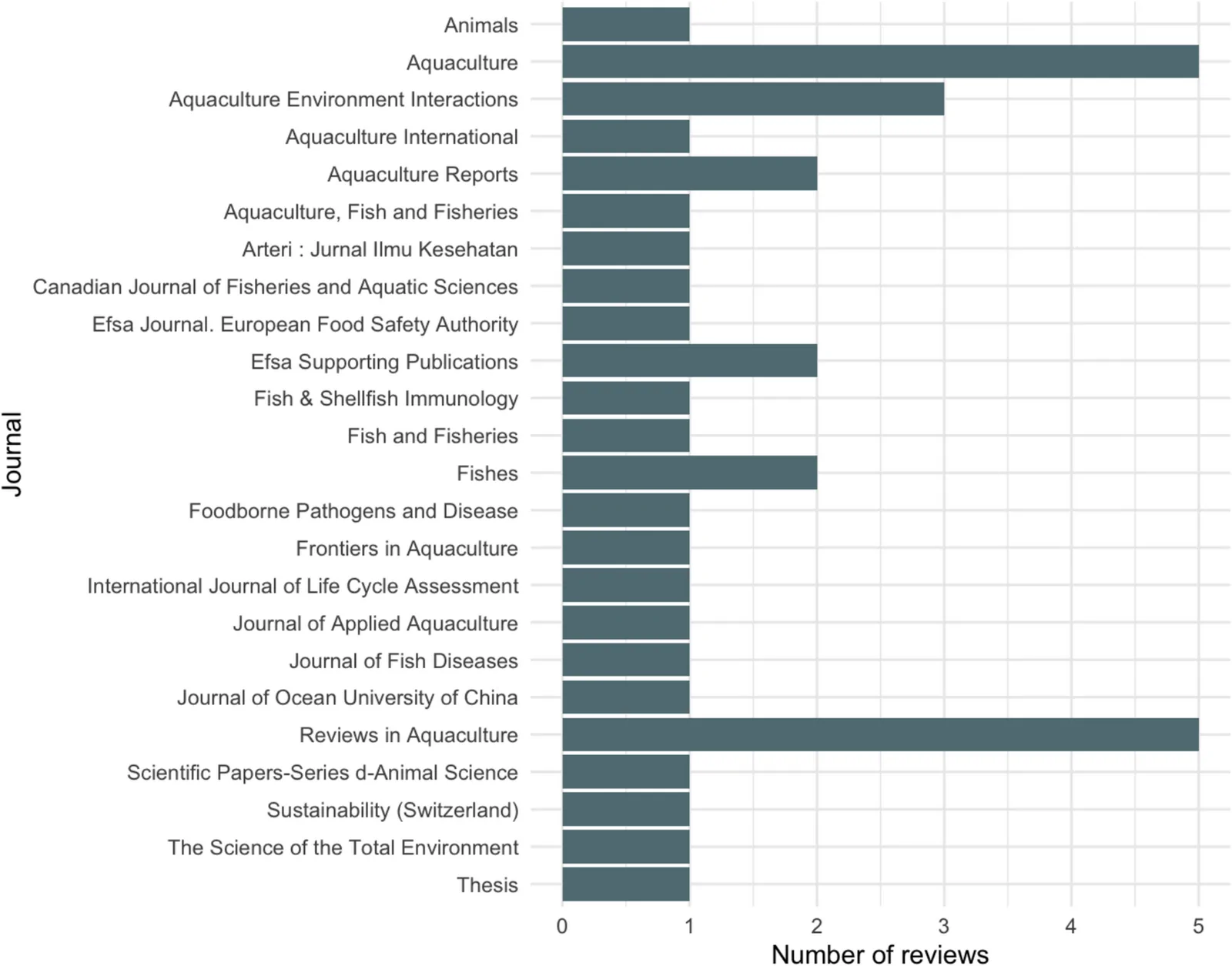
Journals featuring the included reviews by review count

The academic journals “Aquaculture” and “Reviews in Aquaculture” were the two most commonly represented outlets, each with 5 reviews (14%) (Fig. 3). One other journal, “Aquaculture Environment Interactions”, featured 3 reviews (9%), whilst other journals each featured 1 to 2 reviews (3–6%).

**Fig. 4:**
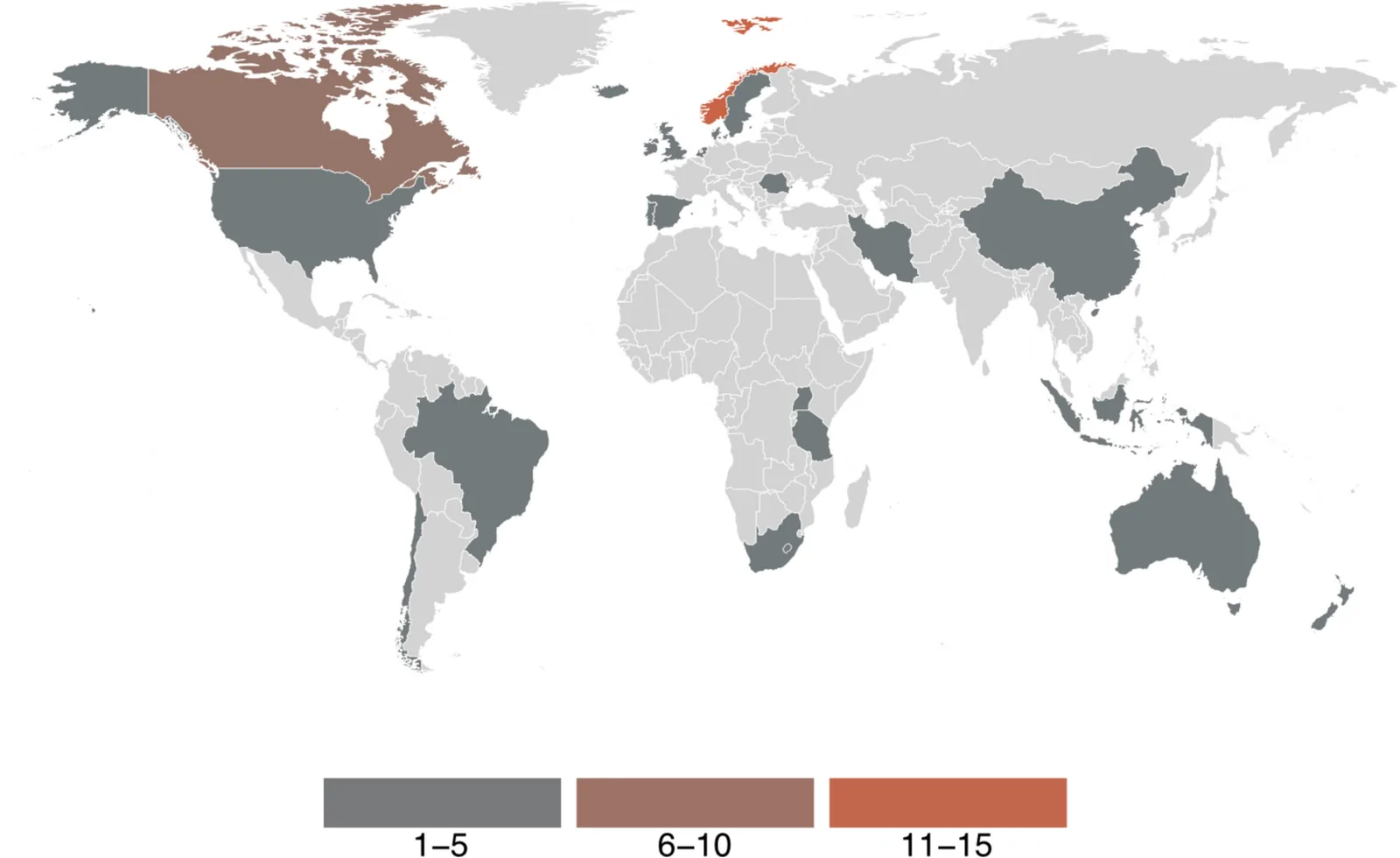
Included reviews across affiliation countries of all authors (total author n = 73)

Authors most commonly hailed from the major salmon-producing nations (Norway, n = 18, 25%; Canada, n = 8, 11%; United Kingdom, n = 4, 5%), with other countries represented by three (4%) or fewer authors (Fig. 4).

**Fig. 5:**
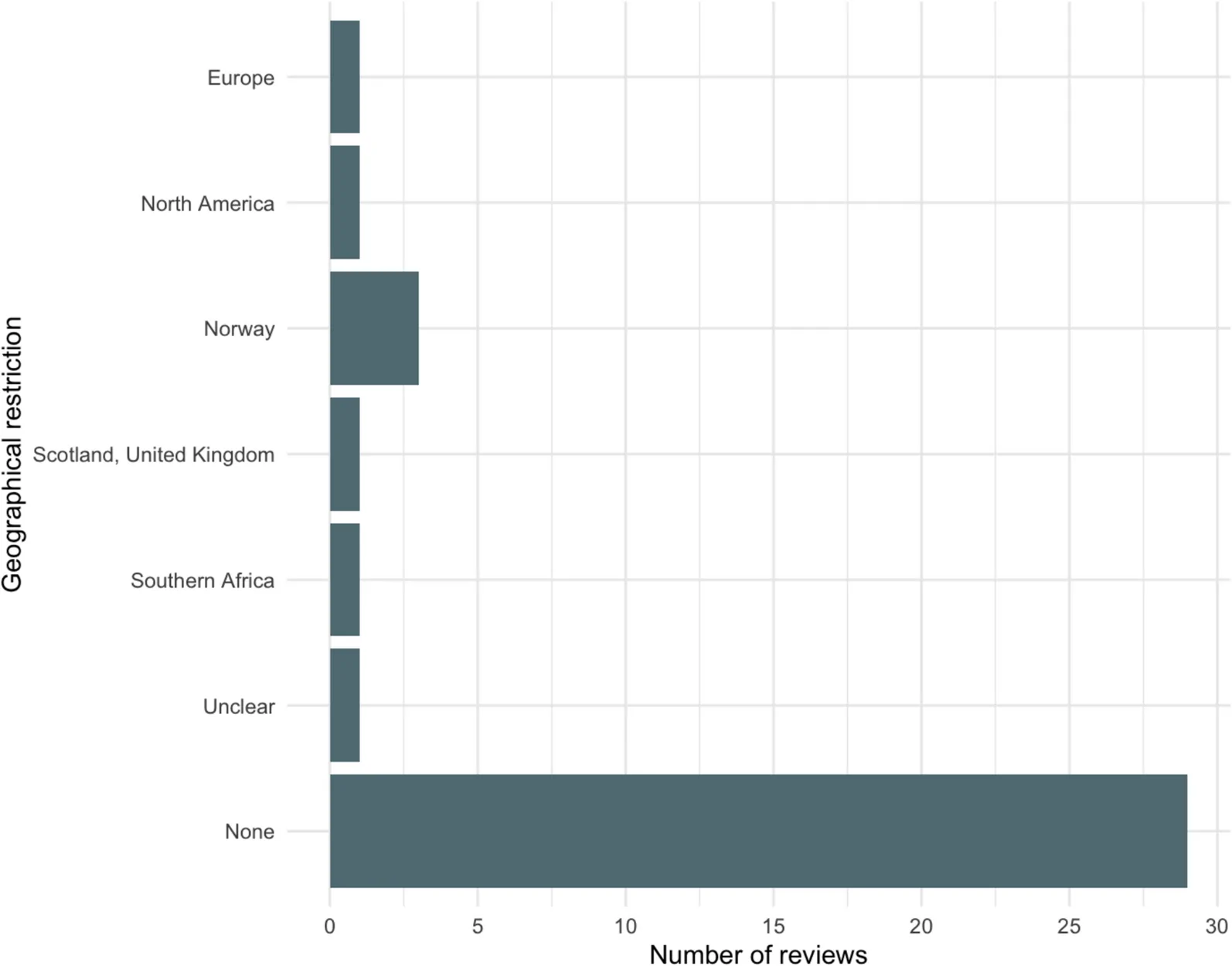
The number of reviews by geographical focus

Most reviews were generalised across producing countries or regions without geographic restriction (Fig. 5), with three reviews focusing on Norway specifically, and Southern Africa, the UK (Scotland), North America and Europe each the focus of 1 review.

**Fig. 6:**
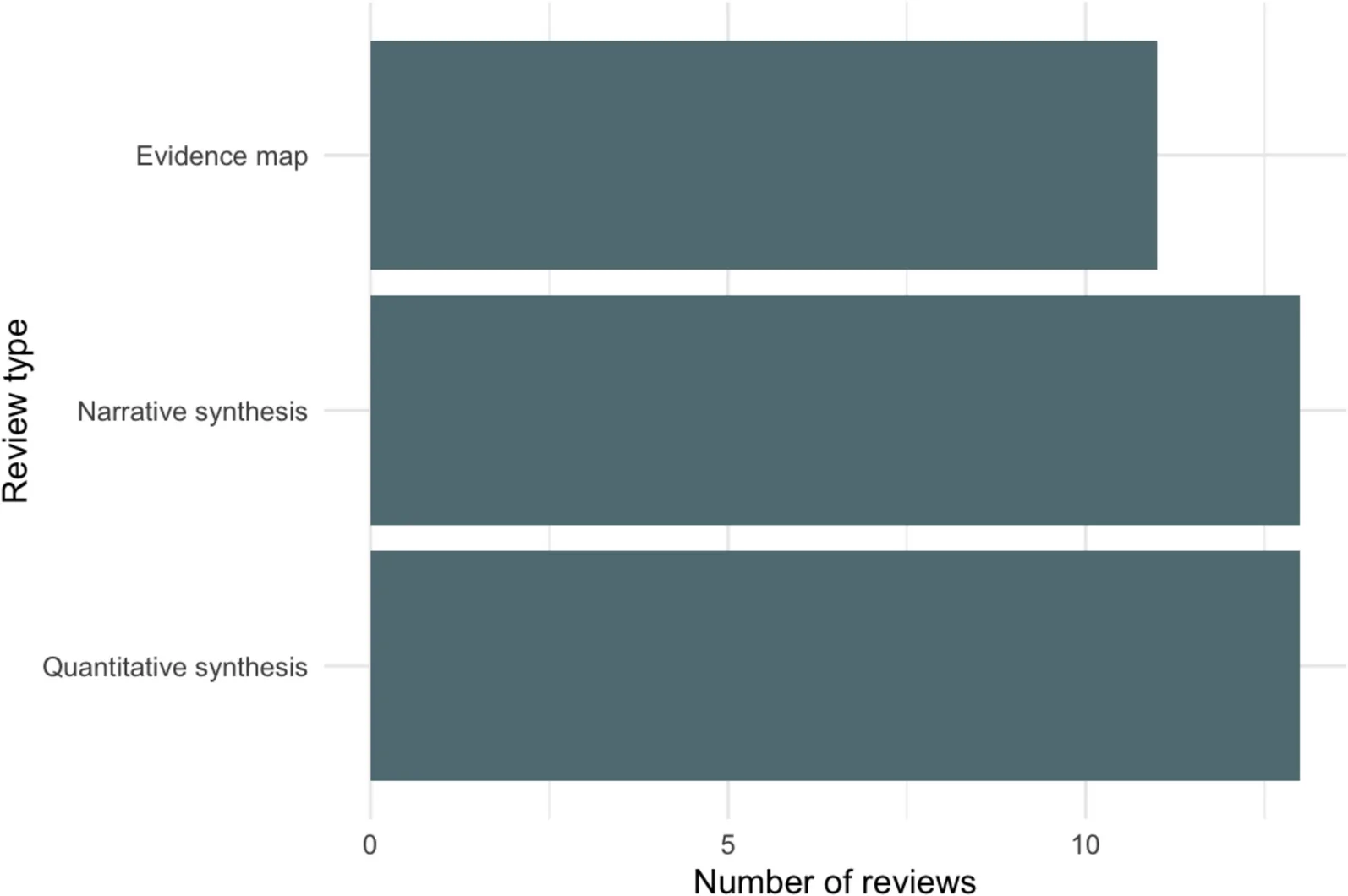
Number of reviews by synthesis type

The majority of reviews were narrative syntheses (n = 13, 35%) or quantitative syntheses (involving some form of meta- or other analysis, n = 13, 35%), with 11 reviews (30%) that aimed to map the evidence base without fully synthesising study findings (Fig. 6).

**Fig. 7:**
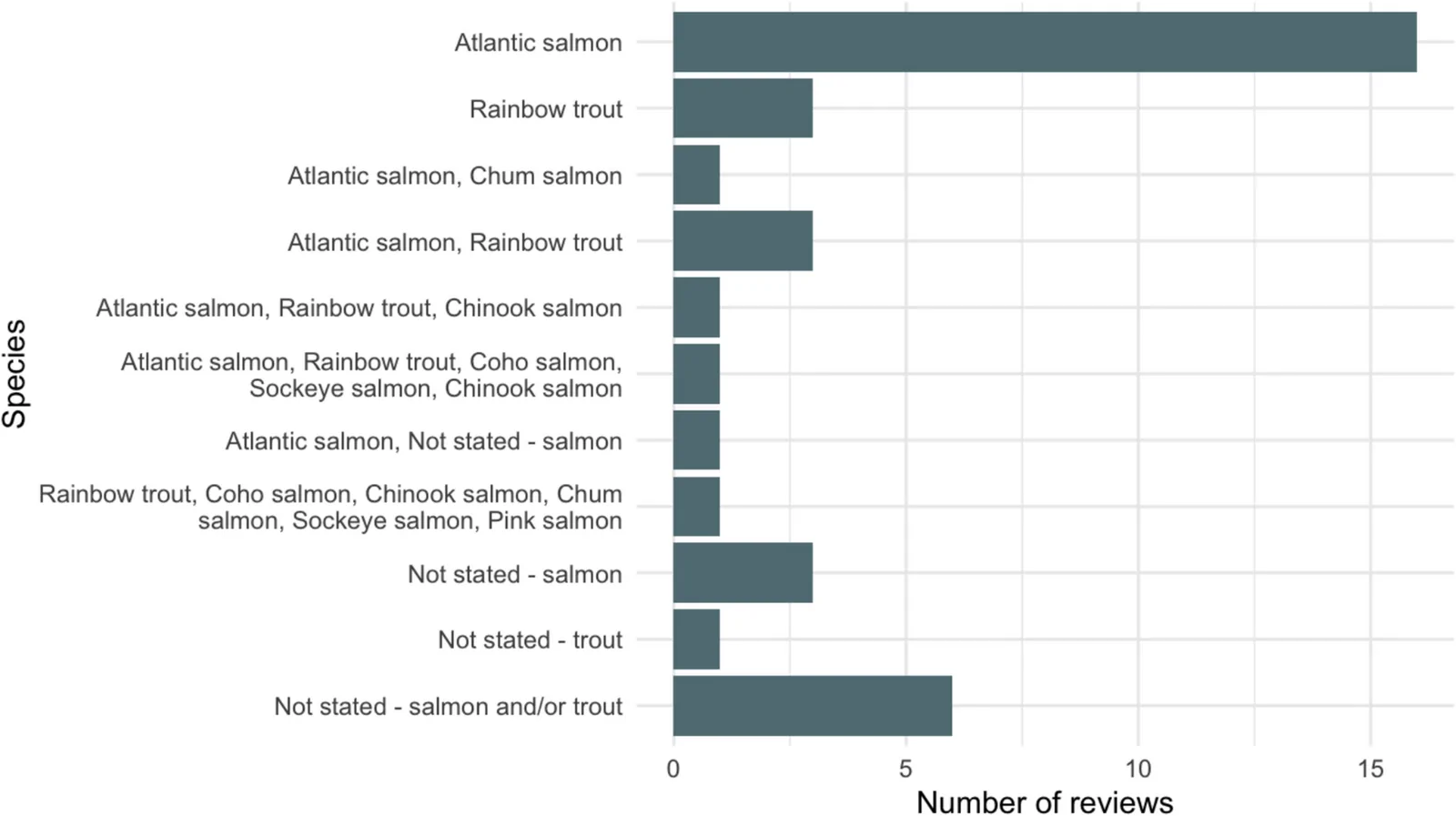
Study species represented across included reviews

In line with their economic importance, Atlantic salmon (*S. salar*, n = 23, 62%) followed by rainbow trout (*O. mykiss*, n = 9, 24%) were the most frequently studied species. Relevance to an unspecified salmon and/or trout species was commonly made (n = 11, 30%), with other salmon species all mentioned fewer than 4 times each. A total of 8 reviews (22%) focused on multiple species (Fig. 7).

**Fig. 8:**
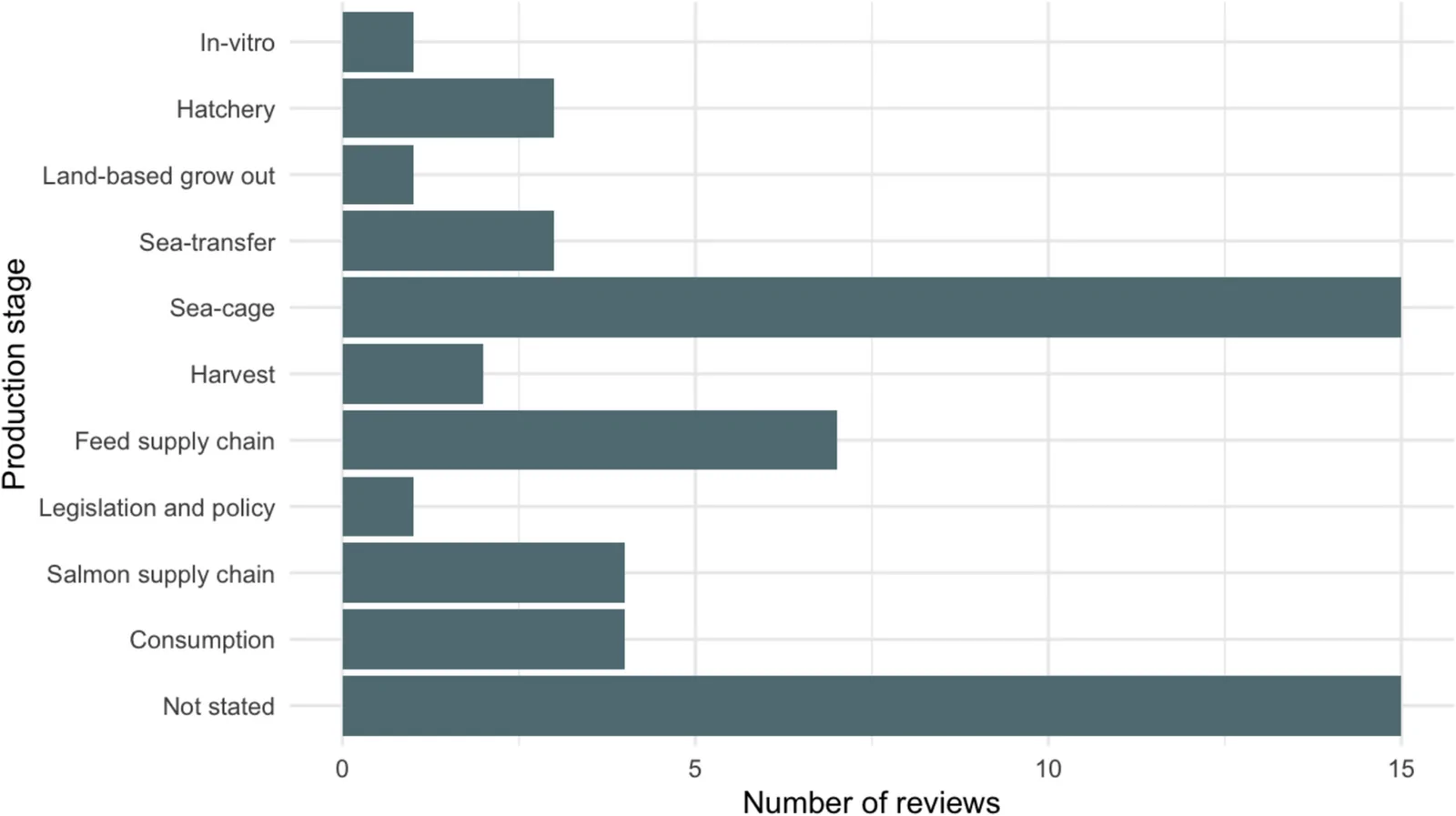
Number of reviews by production stage. Some reviews focused on multiple production stages (categories are not mutually exclusive)

The most common production stage was sea-cage grow-out (n = 15, 41%; Fig. 8), followed by feed supply chains (the focus of several life-cycle analyses, n = 7, 19%). Salmon supply chains and consumption were also represented by multiple reviews (n = 4 and 4, respectively, 11%). Some 15 (41%) reviews did not mention the production stage or were relevant across stages.

**Fig. 9:**
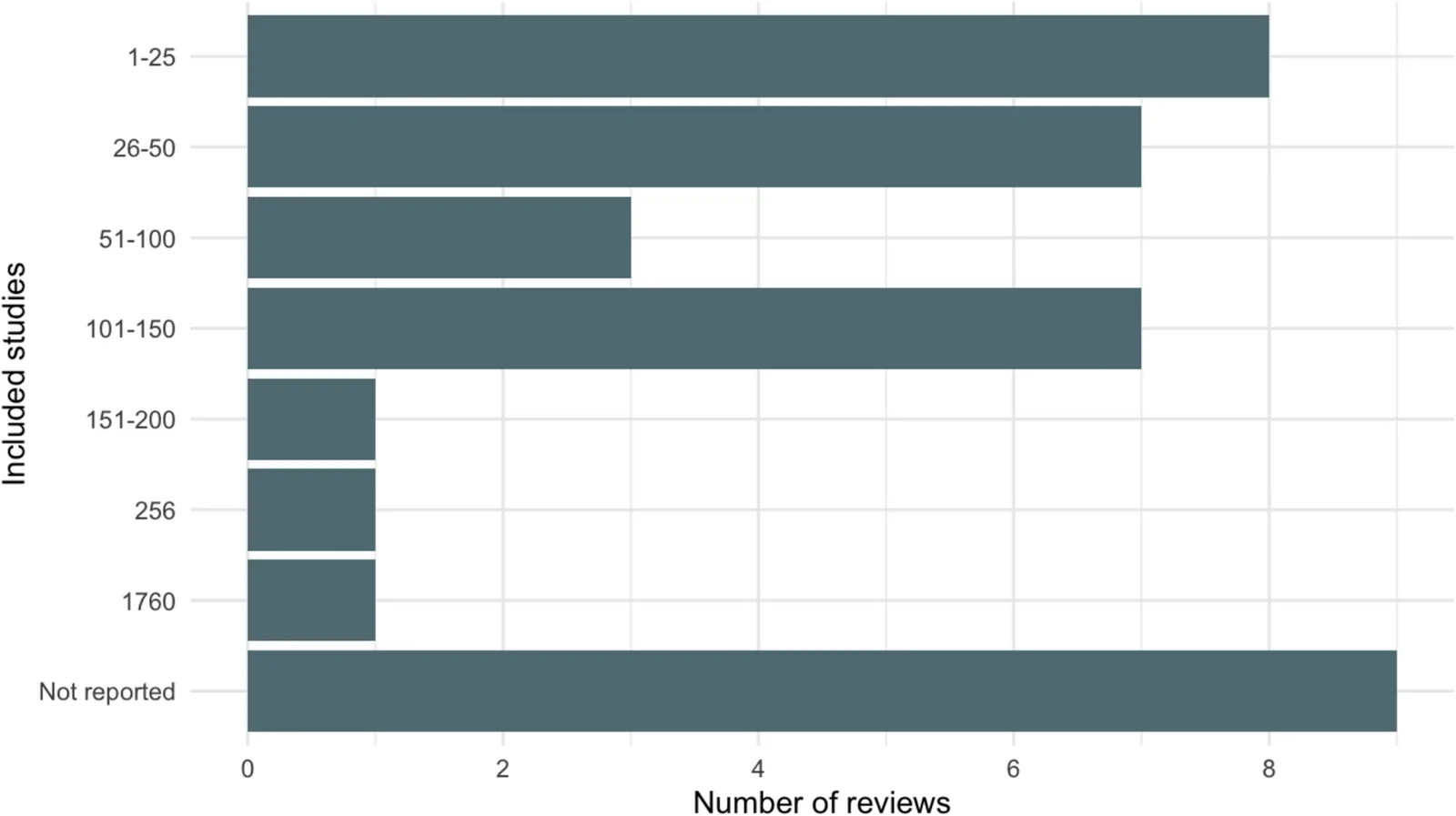
Number of primary studies included in the reviews

The number of included primary studies in each review was generally low, with 15 reviews including 50 or fewer studies (41%) (Fig. 9). Seven reviews included between 101 and 150 studies. One review, a scoping review, included a much larger number of studies (1,760 included studies). A total of 9 reviews failed to report the number of included studies.

**Fig. 10:**
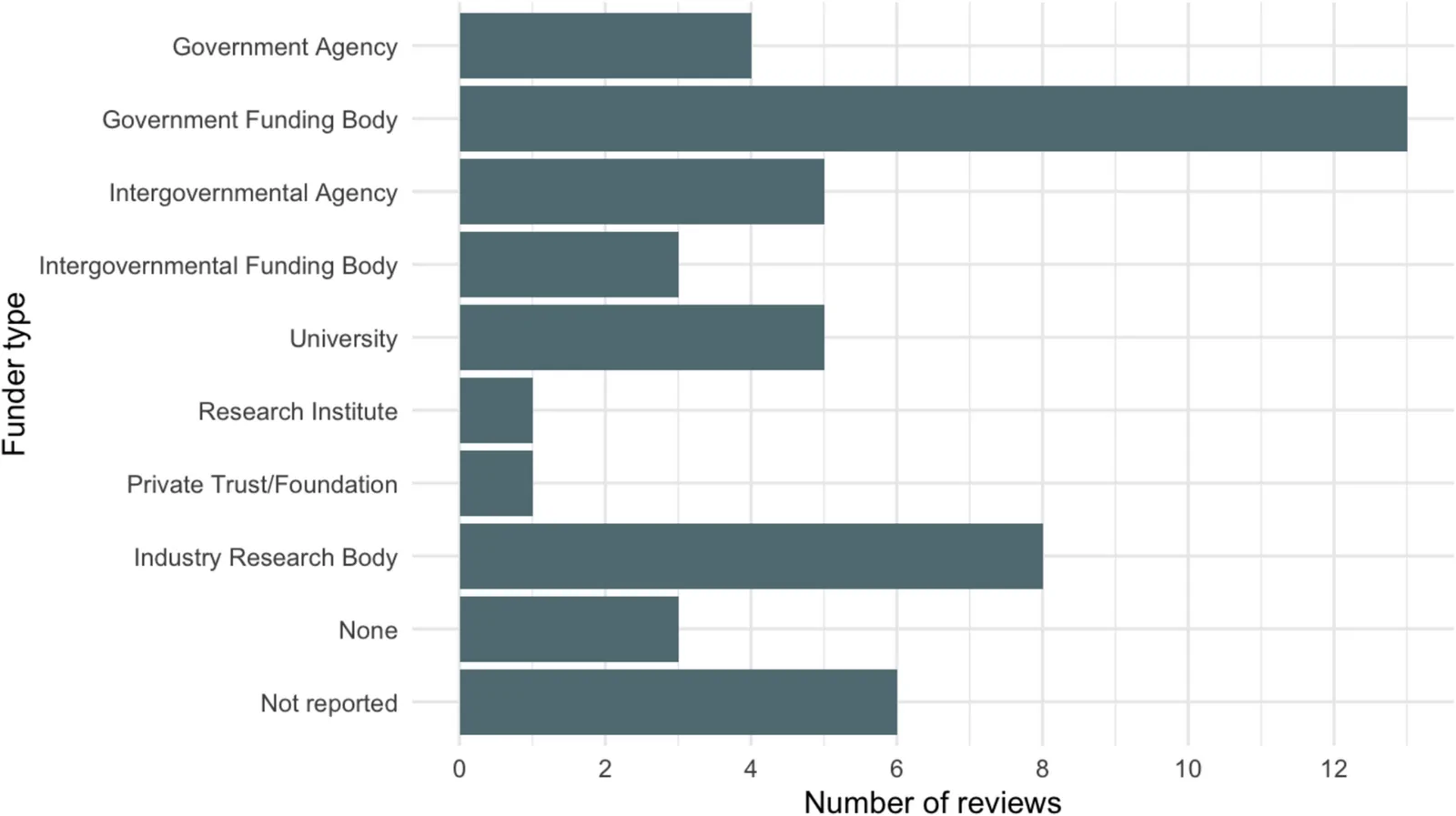
Review funder type across the included reviews. Some reviews had multiple funders (categories are not mutually exclusive)

The most common funder for the reviews was a government funding body (n = 13, 35%), followed by an industry research body (n = 8, 22%), while funding was not reported for six reviews (16%). Furthermore, an analysis of co-author affiliations demonstrated that 7 reviews (19%) included industry partners as co-authors, with 9 total industry partners listed as co-authors across these articles. Companies listed as co-authors were: AquaGen AS, Bakkafrost Scotland, BioMar Group, Blue Genomics Chile, Cermaq Finnmark AS, Marine Prospects AS, Mowi Scotland, Nordlaks Oppdrett AS, and Scottish Sea Farms Ltd (Fig. 10).

**Fig. 11:**
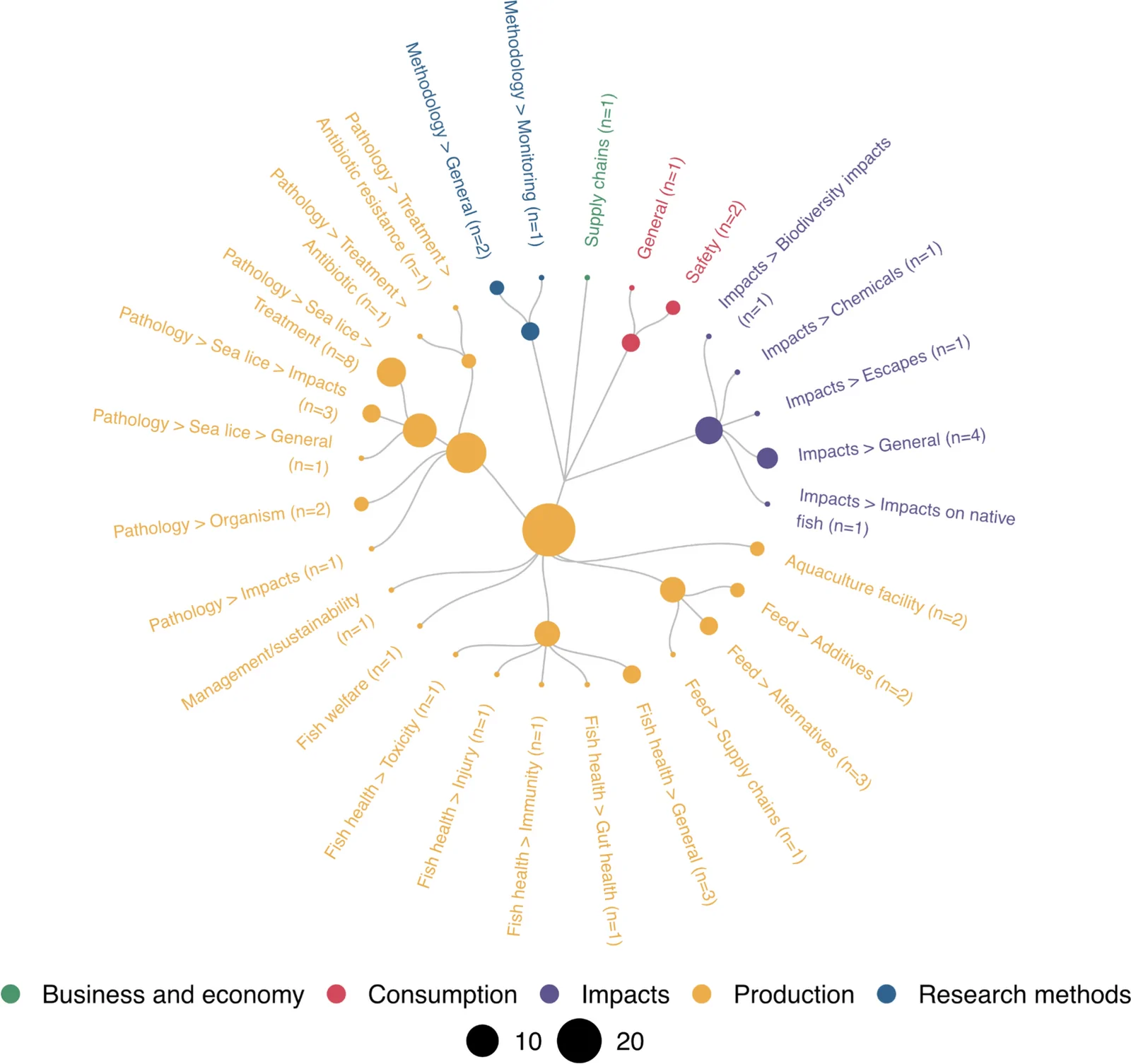
Study subjects referenced within the included reviews. Legend and colour represent broad topic. Coding is provided in a hierarchical structure: [Subtopic] > [Feature] n = [number of reviews]. Multiple outcomes can exist per review, total outcome n = 49

Production subjects were the most commonly investigated (Fig. 11), with pathology and sea lice specifically contributing the greatest research attention. Environmental impacts and research methodology were also described across multiple reviews.

**Fig. 12:**
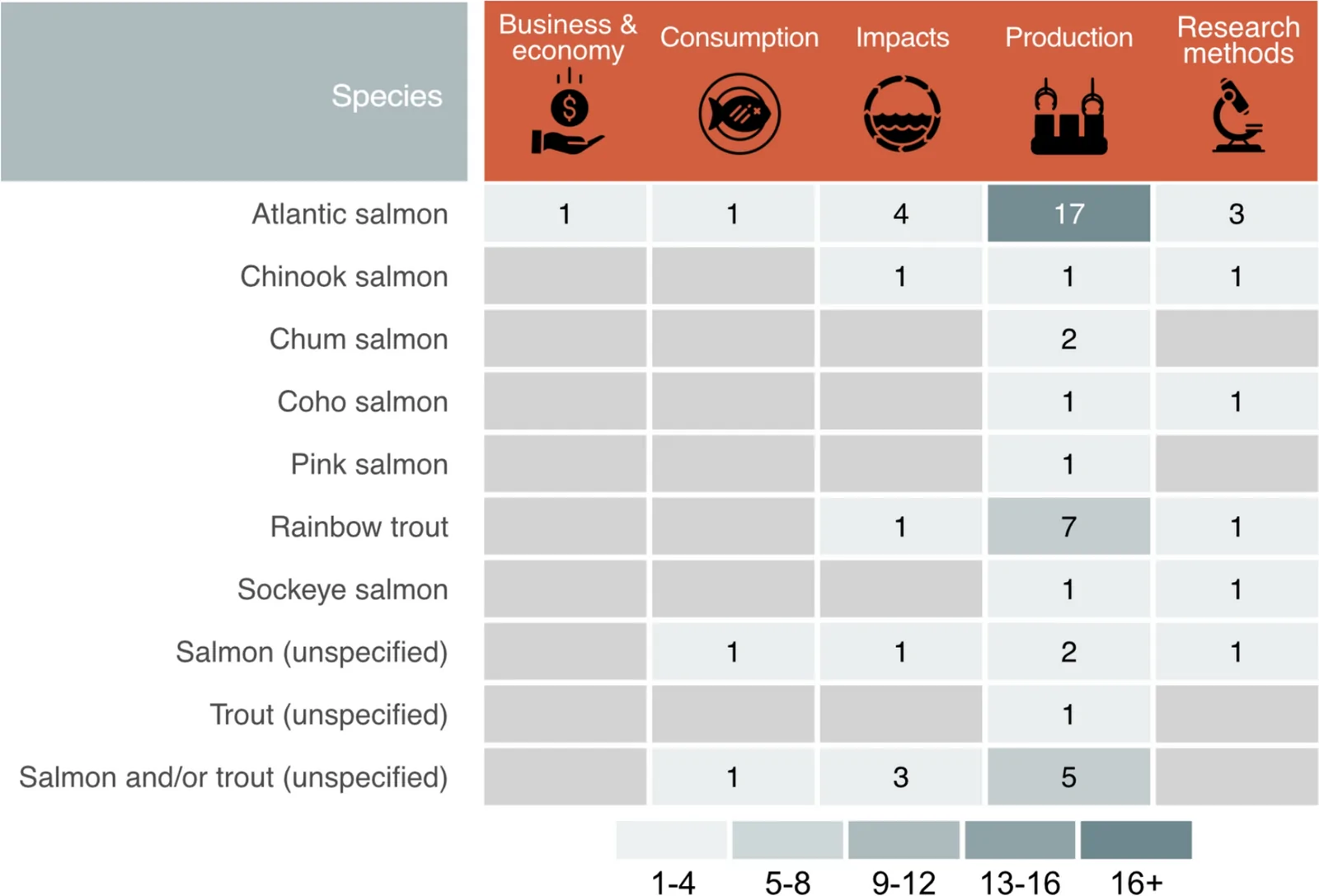
Study subject by species in the included reviews

The majority of reviews focused on Atlantic salmon (n = 17, 46%) and rainbow trout (n = 7, 19%) production (Fig. 12). Beyond production, other species were represented poorly with just single studies across other categories of subjects.

**Fig. 13:**
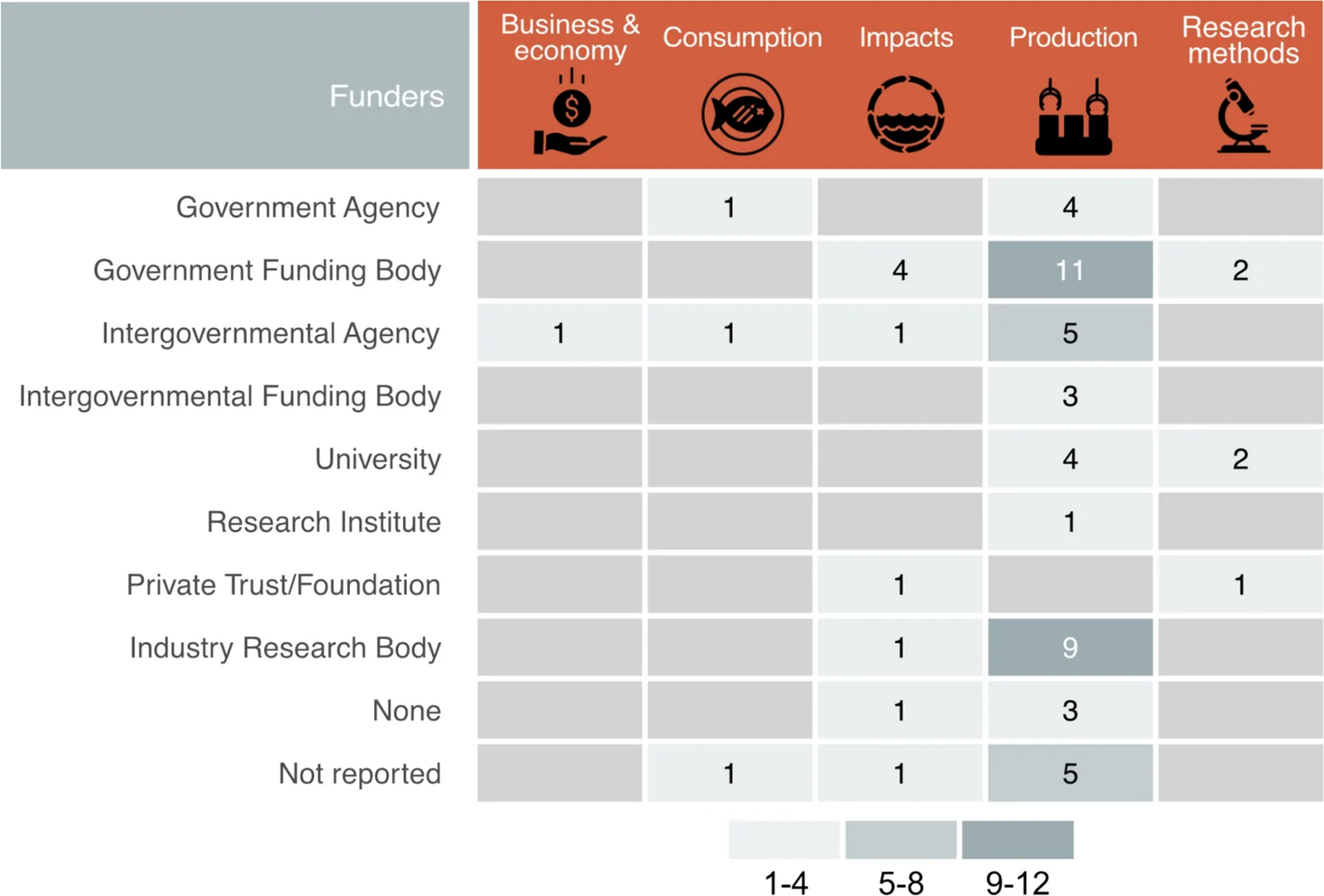
Review funder by outcome focus of the included reviews

As described above, most research focused on production outcomes. Figure 13 demonstrates that government funding bodies supported 11 of these reviews (30%), industry research bodies supported nine (24%) and intergovernmental agencies supported five (14%), while a wide diversity of funders supported the remaining reviews (e.g. universities and a research institute). Other funders also contributed to research into impacts and research methods, with government funding bodies supporting four reviews on impacts (11%) and two on research methods (5%). Industry also supported one review on impacts (3%).

### Mapping the quality of included reviews

**Fig. 14:**
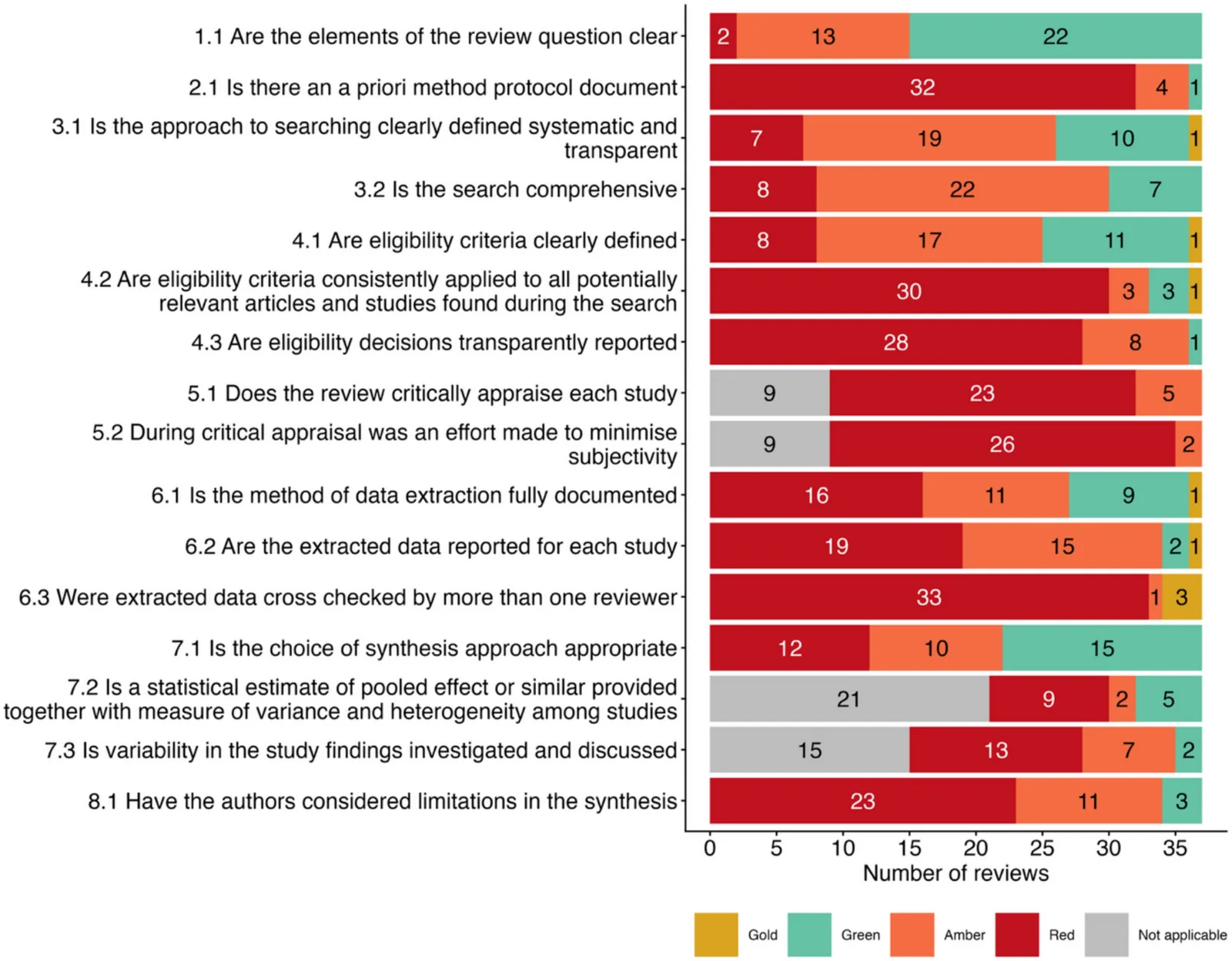
Summary of the judgements for review reliability for each CEESAT question

**Fig. 15:**
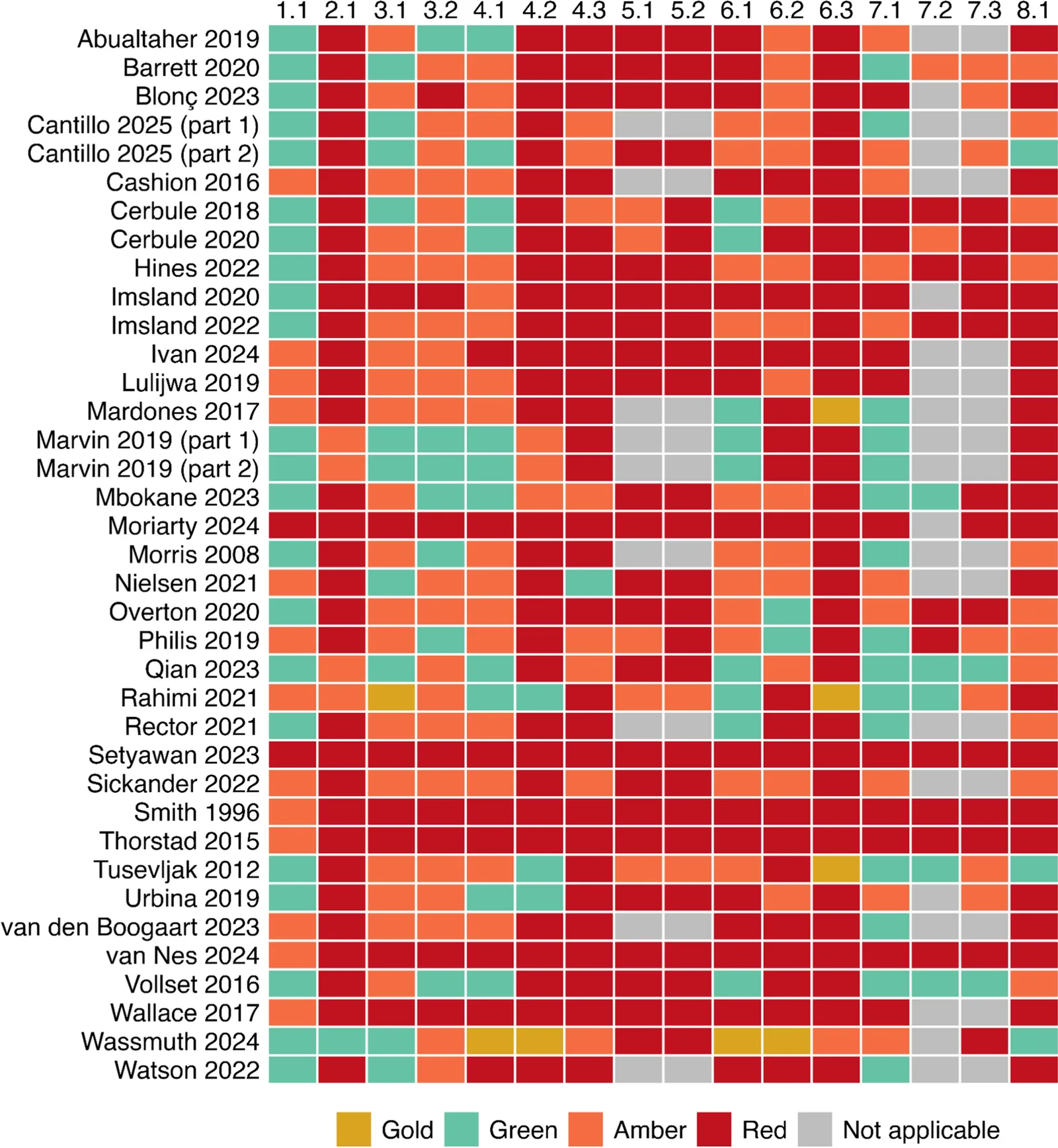
CEESAT scores and overall reliability judgement score for all included reviews

We have summarised the results of our study validity assessment in Fig. 14, and provide judgements for all questions across each review in Fig. 15. In the following text, we describe the status of the evidence base in terms of rigour across each of the 8 steps of the review process and the 16 questions included in the CEESAT appraisal.

In summary, only four reviews were judged to be of moderate rigour (11%), whilst none obtained a high rigour rating. In contrast, a total of nine reviews were judged to be of exceptionally low rigour (24%), and the remaining 23 reviews were deemed to be of low rigour (62%).

Our analysis shows that reviews in the field were conducted and reported poorly, with 285 “Red” judgements showing considerable methodological failings, and only 8 “Gold” judgements of high rigour. Grey cells indicate ‘not applicable’ for cases where evidence mapping or narrative synthesis was performed.

### The question (Q1.1)

Just over half (n = 21, 57%) of the reviews provided a question that was clear and well-defined, although no reviews used an established framework for systematic review questions, such as PICO/PECO. Some 35% of the reviews (n = 13) described their question vaguely in terms that allowed the broad concepts to be understood, but two reviews failed to define their question or scope.

### The protocol (Q2.1)

Only one review made reference to a protocol (whether a priori or not), with a further three reviews (8%) providing sufficient detail for the methods to be ex post inferred. However, the majority (86%, n = 32) failed to make use of a protocol document outlining the planned methods and also failed to provide sufficient methodological detail to allow replicability.

### The search (Q3.1, Q3.2)

Some nine reviews (24%) provided search details in a way that was replicable, with a further 19 (51%) giving some information about their searches. However, seven reviews gave no details of their search methods at all, with just one review providing these methods in an a priori protocol. Similarly, only six reviews (16%) searched across an appropriate number of resources (i.e. more than two bibliographic databases and searching for grey literature), with the remainder either searching only one or two databases or omitting grey literature (59%, n = 22), or providing no information about where searches were performed (22%, n = 8).

### Screening for eligibility (Q4.1, Q4.2, Q4.3)

The criteria for primary study inclusion were clear (“Green”, n = 10) or inferable (“Amber”, n = 17) for the majority of reviews (73%), with eight reviews failing to define criteria at all and one review pre-specifying inclusion criteria in an a priori protocol. However, consistency checking or dual screening was rarely performed, with the majority (81%, n = 30) giving no details of any cross-reviewer verification. Reporting of screening decisions was particularly poor across reviews, with 73% (n = 27) giving no details of the number of screened or included studies and a further 22% (n = 8) giving both the number of exclusions and a list of included studies.

### Critical appraisal of study validity (Q5.1, Q5.2)

For narrative and quantitative reviews, critical appraisal should have been performed (it is not necessary for evidence maps). For these reviews, the majority (82%, n = 23) failed to assess any aspect of risk of bias or aspects of study validity. In only two cases were any methods employed to minimise subjectivity during critical appraisal.

### Data extraction (Q6.1, Q6.2, Q6.3)

Reviews performed relatively poorly for formalised data extraction. Some 43% of reviews (n = 16) failed to describe the methods of data extraction in any way, with a further 30% (n = 11) providing minor details that precluded replicability. Slightly fewer studies reported the extracted data, with 49% (n = 18) reporting no data at all, and 41% (n = 15) providing only selected or limited data. One study notably described data extraction methods and presented the extracted data in full detail. In three reviews, data were reported to have been extracted by more than one reviewer, but the majority of studies failed to describe this cross-checking (presumably because it did not occur).

### Synthesis (Q7.1, Q7.2)

Some of these questions in this CEESAT category only apply to quantitative syntheses (7.2) and narrative syntheses (7.3), and not evidence maps. In general, narrative syntheses and evidence maps scored relatively well, although no reviews had chosen and justified their synthesis methods in advance (in an a priori protocol). Some 12 reviews scored badly due in part to the reliance on vote-counting methods or other inappropriate quantitative synthesis practices. Of the quantitative syntheses, around 50% (n = 10) of the reviews scored poorly for good statistical practices (i.e. providing pooled estimates of effects along with a measure of variance and heterogeneity). Similarly, a third (n = 13) of the quantitative and narrative syntheses failed to discuss or investigate variability in the findings extracted from primary studies.

### Limitations (Q8.1)

Few reviews discussed their limitations, with less than 10% of reviews (n = 3) explicitly referencing any limitations of the review or the evidence base. No reviews had an explicit section dedicated to the limitations of both that discussed all major potential limitations to their methods or the evidence base (corresponding to a “Gold” judgement).

### Narrative synthesis

We summarise only the findings of the four reviews judged to be of moderate rigour during study validity assessment i.e. [13, 15, 37, 44]. We do not discuss the findings from reviews rated as low or exceptionally low rigour, as we consider them unreliable and prone to a high risk of bias based on their methodological inadequacies.

### Moderately rigorous review findings

Several key themes emerge from these reviews, including the environmental performance of alternative feed proteins, the comparative sustainability of different production technologies, the risks associated with escaped farmed salmon, and the effectiveness and sustainability of salmon lice control methods.

Cantillo & Deshpande [13] systematically reviewed recent literature (2015–2024) to identify alternative protein sources for Atlantic salmon aquaculture and evaluate their environmental performance, focusing on carbon footprint hotspots and strategies for improvement. They synthesised 91 primary studies, highlighting that while research on insect and single-cell proteins is growing, findings on their carbon footprints were highly variable (ranging from 0.1 to 132 kg CO₂/kg protein), largely due to inconsistent assumptions, system boundaries, and LCA methodologies. This limits the comparability of results and hinders robust conclusions on climate benefits, though energy and substrate production are consistently cited as key hotspots.

Philis et al. [44] reviewed and compared methodological choices, inventories, and modelling protocols in salmonid Life Cycle Assessment (LCA) studies, and performed a cross-study statistical analysis of Life Cycle Impact Assessment and Feed Conversion Ratios (FCR) results to assess comparability, identify discrepancies, and evaluate the need for methodological improvements. The authors report that methodological inconsistencies across 24 LCA studies prevent definitive comparisons of production systems. Open-net pens showed lower energy demands but higher eutrophication potential, while Recirculating Aquaculture systems exhibited higher carbon footprints. FCR were commonly referenced as key performance indicators, but confounding factors such as feed composition and system conditions limit their utility across contexts.

Morris et al. [37] aimed to review farmed salmon escapes in eastern North America, assessing which rivers have been surveyed, quantifying the proportion of escaped farmed salmon relative to wild populations, and examined temporal trends in their presence. They provide strong observational evidence of the widespread presence of escaped farmed salmon—recorded in 87% of rivers in North America surveyed within proximity to aquaculture sites between 1984 and 2006—but note a lack of standardised monitoring and insufficient genetic or population-level impact assessments. While negative effects such as genetic introgression and disease transmission were well documented in individual studies, broader conclusions were hampered by fragmented and short-term datasets.

Cerbule [15] systematically reviewed the effectiveness of the three most widely used salmon lice treatments in Atlantic salmon aquaculture: chemical treatments, cleaner fish, and warm water treatment (thermolicing). The study identified similar weaknesses in the evidence on lice control, with most data focusing on increasingly ineffective chemical treatments, while cleaner fish show promise but lack long-term, regionally generalisable studies. Warm water treatments were underrepresented in the literature, and little is known about their welfare implications or environmental trade-offs.

Across all reviews, the evidence base is constrained by uneven research coverage, methodological heterogeneity, and poor integration of environmental externalities. There is a strong consensus on the need for harmonised frameworks, uncertainty analysis, and more robust comparative research to inform policy and improve the sustainability of salmon aquaculture.

### Limitations of the review

Our review is not without its limitations. We have not searched for grey literature beyond theses and dissertations, although we argue that our scope is restricted to academic publications. We have used a relatively inclusive definition of “systematic reviews”, including anything that either self-identifies as such, or includes some aspect of explicit methodology. We did this purposefully to avoid including primers or overviews that did not set out to be systematic, but our included set may overlap with excluded articles that do not refer to themselves as systematic. However, we believe our searches and screening were comprehensive enough that the risk of missing relevant reviews is very low. We will also not have included reviews focused on topics relevant to but not explicitly referencing salmon aquaculture—for example, reviews of sea lice treatment effectiveness. However, we believe it unlikely that these reviews will have neglected to include a reference to their core subject, salmon.

We also searched only English language databases. We acknowledge that evidence syntheses may exist in non-English language resources and journals, and further work should be undertaken to identify and appraise these, should they exist. However, our analysis is a methodologically robust assessment of the published English-language secondary research literature pertaining to salmon aquaculture, and our results paint a bleak picture of the state of the evidence identified.

We draw on selected grey literature and journalistic sources to contextualise gaps in the peer-reviewed evidence base, particularly where issues such as industry practices and governance are underrepresented in academic research. While peer review is not a guarantee of evidential quality, these sources have not been subject to the same formal academic system of appraisal as the included systematic reviews and should therefore be interpreted as contextual rather than synthesised evidence. It should be noted, however, that investigative journalism is rigorously reviewed and vetted to ensure a legally defensible burden of proof, and responsive to critique and retraction at much faster speeds than in the academic literature.

## Conclusions

This overview of reviews identified a relatively small body of systematic review evidence on salmon aquaculture, with just 37 reviews meeting the inclusion criteria. While a larger number of literature reviews exist, many do not employ systematic methods, highlighting a disconnect between the apparent interest in evidence synthesis and the application of rigorous methodologies. Given the volume of primary research on salmon farming, the limited number of robust quantitative syntheses is notable, particularly for well-studied topics such as sea lice and fish health. At the same time, there is a clear lack of synthesis on environmental and socio-economic impacts, reflecting broader gaps in the primary evidence base.

The included reviews were generally of low methodological quality, with no review achieving a high reliability rating. Common limitations included the absence of a priori protocols, poor reporting of search and screening methods, lack of critical appraisal, and inappropriate synthesis approaches such as vote counting. These findings suggest that the current body of evidence syntheses provides a weak and potentially biased foundation for decision-making.

### Implications for policy and management

Decision-makers should interpret existing evidence syntheses on salmon aquaculture with caution, given the generally low methodological rigour and high risk of bias identified across reviews. Apparent consensus or patterns in the literature may reflect methodological limitations rather than robust underlying evidence. As such, reliance on individual reviews, particularly those lacking transparent methods or critical appraisal, may lead to misleading conclusions.

Furthermore, the current evidence base is heavily skewed towards production-related outcomes, with limited synthesis of environmental and socio-economic impacts. This imbalance should be explicitly recognised in decision-making processes, and absence of synthesised evidence should not be interpreted as evidence of absence. In the context of such uncertainty, precautionary and transparent approaches to decision-making are warranted.

### Implications for research

There is a clear need for a substantial improvement in the methodological rigour of evidence syntheses in the field of salmon aquaculture. Future reviews should adhere to established best practices, including the use of a priori protocols, comprehensive and reproducible search strategies, transparent screening and data extraction methods, and formal critical appraisal of included studies.

In addition, there is an urgent need to expand the scope of evidence synthesis beyond production-focused topics to include environmental and socio-economic impacts of salmon farming and its associated supply chains. Addressing these gaps will be essential for supporting balanced and evidence-informed policy and management decisions.

Finally, greater independence and transparency in research funding and authorship are needed to reduce potential biases in the evidence base and to ensure that future syntheses provide a reliable foundation for decision-making.

## Supplementary Information

Additional file 1.

Additional file 2.

Additional file 3.

Additional file 4.

Additional file 5.

Additional file 6.

## Data Availability

Data is provided within the manuscript or supplementary information files.
