# Supplementary material for: What evidence exists on salmon farming? An overview of reviews
Source: Environ Evid. 2026 Jul 15;15:12. doi: 10.1186/s13750-026-00391-y (PMC13651248; doi:10.1186/s13750-026-00391-y)
Supplement: Supplementary file 1 [file 13750_2026_391_MOESM5_ESM.docx]

# **Additional File 5: Benchmark list**

1. Bjarnason, A. and Magnusdottir, K.S., 2021. The Salmon Sea Fish Farming Industry in Iceland. *A review. Fish Aqua J*, *10*(2).

Iceland has for a long time been renowned for its rich fishing grounds and prolific fisheries sector. Salmon sea fish farming in Iceland, as we know it today, is however a relatively young industry which goes back only few years. It has however, already experienced strong growth in investment, production, exports revenue and is a provider of employment in rural areas in Iceland. Operating licences issued for salmon farming in sea cages in Iceland, amount in total to less than 60 thousand tons. The four biggest companies in the industry which control nearly all of the operating licences issued in Iceland, are majority owned or fully owned by Norwegian investors and existing companies in the Norwegian salmon industry. With one or two exceptions, for some reason, companies in the Icelandic fisheries and fish processing sector, do not participate as investors and shareholders in the build-up of the salmon sea fish farming industry in Iceland, and therefore, have not added salmon into their fish production.

2. Bravo, S. and Treasurer, J., 2023. The management of the sea lice in Chile: A review. *Reviews in Aquaculture*, *15*(4), pp.1749-1764.

Sea lice infestations have the largest economic and biological impact on salmon farming in Chile, similar to that seen in salmon‐producing countries in the northern hemisphere. Caligus teres infesting coho salmon (Oncorhynchus kisutch) was first recorded in 1981, but Caligus rogercresseyi has been the predominant species infesting Atlantic salmon (Salmo salar) and rainbow trout (Oncorhynchus mykiss) from 1997. In contrast, coho salmon has been shown to be resistant to this sea louse species. Regulations to establish integrated pest management of sea lice were implemented by the Chilean authority in 2007. However, to date, pharmacological medicines have been the main tool used by the salmon farming industry in Chile to manage sea lice, although non‐pharmacological treatments have been available since 2016. Production of farmed salmon reached 978.274 tons in 2021 and 7.72 g of medicines was prescribed per ton of harvested salmon. The highest volumes used in that year were Azamethiphos (47%) and hexaflumuron (45.5%), in addition to 5532 tons of hydrogen peroxide. This review updates the current management of sea lice in Chile and also summarizes the biological knowledge of this important parasite.

3. Duman, M., Altun, S., Saticioglu, I.B. and Romalde, J.L., 2023. A review of bacterial disease outbreaks in rainbow trout (Oncorhynchus mykiss) reported from 2010 to 2022. *Journal of Fish Diseases*.

Outbreaks of bacterial infections in aquaculture have emerged as significant threats to the sustainable production of rainbow trout (Oncorhynchus mykiss) worldwide. Understanding the dynamics of these outbreaks and the bacteria involved is crucial for implementing effective management strategies. This comprehensive review presents an update on outbreaks of bacteria isolated from rainbow trout reported between 2010 and 2022. A systematic literature survey was conducted to identify relevant studies reporting bacterial outbreaks in rainbow trout during the specified time frame. More than 150 published studies in PubMed, Web of Science, Scopus, Google Scholar and relevant databases met the inclusion criteria, encompassing diverse geographical regions and aquaculture systems. The main bacterial pathogens implicated in the outbreaks belong to both gram‐negative, namely Chryseobacterium, Citrobacter, Deefgea Flavobacterium, Janthinobacterium, Plesiomonas, Pseudomonas, Shewanella, and gram‐positive genera, including Lactococcus and Weissella, and comprise 36 new emerging species that are presented by means of pathogenicity and disturbance worldwide. We highlight the main characteristics of species to shed light on potential challenges in treatment strategies. Moreover, we investigate the role of various risk factors in the outbreaks, such as environmental conditions, fish density, water quality, and stressors that potentially cause outbreaks of these species. Insights into the temporal and spatial patterns of bacterial outbreaks in rainbow trout aquaculture are provided. Furthermore, the implications of these findings for developing sustainable and targeted disease prevention and control measures are discussed. The presented study serves as a comprehensive update on the state of bacterial outbreaks in rainbow trout aquaculture, emphasizing the importance of continued surveillance and research to sustain the health and productivity of this economically valuable species.

4. Gowen, RJ, 1987. The ecological impact of salmonid farming in coastal waters: a review. *Oceanogr. Mar. Biol. Ann. Rev.*, *25*, pp.563-575.

The composition and quantity of wastes (uneaten food, faecal and excretory material) generated by cage culture of Atlantic salmon and rainbow trout, and the potential impact of this waste on both the marine environment and on production of the fish farm itself are reviewed. A number of compounds are found in fish-farm waste, but organic C, resulting in sediment enrichment, and dissolved N, resulting in hypernutrification of surrounding waters, are the most important. Sediment anoxia, reduction of benthic macrofauna, and eutrophication tend to be localized. Potential negative impacts on salmonid culture include outgassing of H2S and induction of toxic blooms. Careful preselection of sites with good water exchange, reduction of food wastage, and periodic cage redeployment are suggested to minimize environmental damage and negative feedback. Dept. of Biol. Sci., The Univ., Stirling, Scotland. (gsb)

5. Hines, I.S., Marshall, M.A., Smith, S.A., Kuhn, D.D. and Stevens, A.M., 2023. Systematic literature review identifying bacterial constituents in the core intestinal microbiome of rainbow trout (Oncorhynchus mykiss). *Aquaculture, Fish and Fisheries*, *3*(5), pp.393-406.

Fish aquaculture has become the fastest growing sector in global food production. Thus, ensuring the sustainability of aquaculture practices is of the utmost importance. Studies in higher vertebrates (i.e. mammals) have demonstrated the role of the host microbiome in physiological processes from nutrient acquisition to pathogen protection. Therefore, analysis of fish microbiomes is an important factor to consider with regard to overall animal health and welfare. Rainbow trout (Oncorhynchus mykiss) are an economically valued fish cultured worldwide. Several studies have identified microbial constituents inhabiting the intestinal tract of rainbow trout. To better elucidate some of the core constituents of the rainbow trout intestinal microbiome, this systematic literature review analysed the relative abundance results from 25 articles published on the rainbow trout intestinal microbiome from 2017 to 2021. Bacteria classified within the phyla Firmicutes and Proteobacteria were observed in every study. At the family level, Lactobacillaceae was consistently observed. Additionally, bacteria in the Actinobacteria, Bacteroides, and Tenericutes phyla were identified in at least 50% of the studies. Interestingly, Mycoplasma spp. were occasionally the most dominant organisms present in the microbiome. Overall, the results here identify bacteria that are commonly found members of the rainbow trout intestinal microbiome.

6. Tuševljak, N., Rajić, A., Waddell, L., Dutil, L., Cernicchiaro, N., Greig, J., Wilhelm, B.J., Wilkins, W., Totton, S., Uhland, F.C. and Avery, B., 2012. Prevalence of zoonotic bacteria in wild and farmed aquatic species and seafood: a scoping study, systematic review, and meta-analysis of published research. *Foodborne Pathogens and Disease*, *9*(6), pp.487-497.

Increased reliance on seafood has brought to light concerns regarding food safety, but the information to inform risk assessment or surveillance needs is lacking. A scoping study (ScS) was conducted to characterize published research investigating selected zoonotic bacteria and public health topics in various wild and farmed aquatic species and seafood. This was followed by a systematic review (SR) on selected bacteria (Aeromonas spp., generic Escherichia coli, Salmonella spp., and Vibrio spp.) and aquatic species (clams, mussels, oysters, salmon, and shrimp [including prawn]); a meta-analysis (MA) was conducted only at the retail level due to considerable variability among various pathogen/seafood combinations. The ScS revealed the most frequently investigated themes were farm-level prevalence and intervention research for Vibrio spp. and Aeromonas spp. Antimicrobial use (AMU) and the association between AMU and antimicrobial resistance were rarely investigated. The SR indicated a consistent lack of reporting regarding study methodology and results, precluding the use of many studies in and full benefits of MA. MA of Aeromonas, E. coli, and Salmonella prevalence in retail salmon resulted in pooled estimates of 13% (6–-27%), 2% (0.1–-11%), and 1% (0–5%), respectively. When MA of pathogen/seafood combination resulted in statistically significant heterogeneity (p<0.1), median/range were reported at the region level. The results from our ScS, SR, and MA could be used for better design of future bacteriological surveys of seafood and as inputs for risk assessments or surveillance initiatives in this field.
